# In Situ Photoacoustic Study of Optical Properties of P-Type (111) Porous Silicon Thin Films

**DOI:** 10.3390/nano11051314

**Published:** 2021-05-17

**Authors:** Cristian Felipe Ramirez-Gutierrez, Ivan Alonso Lujan-Cabrera, Cesar Isaza, Ely Karina Anaya Rivera, Mario Enrique Rodriguez-Garcia

**Affiliations:** 1 Cuerpo Académico de Tecnologías de la Información y Comunicación Aplicada (TICA), Universidad Politécnica de Querétaro, El Marqués, Querétaro 76240, Mexico; cesar.isaza@upq.mx (C.I.); karina.anaya@upq.mx (E.K.A.R.); 2 Ingeniería Física, Facultad de Ingeniería, Universidad Autónoma de Querétaro, Querétaro 76010, Mexico; ilujan12@alumnos.uaq.mx; 3 Centro de Física Aplicada y Tecnología Avanzada, Departamento de Nanotecnología, Universidad Nacional Autónoma de México, Campus Juriquilla, Querétaro 76230, Mexico; marioga@fata.unam.mx

**Keywords:** photonic crystal, cross section, reflectance, refractive index, dispersion

## Abstract

Porous silicon (PSi) on p${}^{++}$-type (111) silicon substrate has been fabricated by electronically etching method in hydrofluoric acid (HF) media from 5 to 110 mA/cm${}^{2}$ of anodizing current density. The problem of determining the optical properties of (111) PSi is board through implementing a photoacoustic (PA) technique coupled to an electrochemical cell for real-time monitoring of the formation of porous silicon thin films. PA amplitude allows the calculation of the real part of the films refractive index and porosity using the reflectance self-modulation due to the interference effect between the PSi film and the substrate that produces a periodic PA amplitude. The optical properties are studied from specular reflectance measurements fitted through genetic algorithms, transfer matrix method (TMM), and the effective medium theory, where the Maxwell Garnett (MG), Bruggeman (BR), and Looyenga (LLL) models were tested to determine the most suitable for pore geometry and compared with the in situ PA method. It was found that (111) PSi exhibit a branched pore geometry producing optical anisotropy and high scattering films.

## 1. Introduction

Porous silicon (PSi) is a complex nanostructured material [1,2] usually obtained by electrochemical etching in hydrofluoric acid (HF) based electrolytes [3]. The most relevant properties of PSi are the tuned refractive index through the porosity and the self-limited reaction that passivate the PSi layers once they are formed, allowing the fabrication of multilayered structures. However, the pore formation mechanism involves several chemical reactions, influenced by intrinsic and extrinsic parameters simultaneously [4,5]. As a result, a diversity of pore morphology and sizes can be obtained.

**Figure 1:**
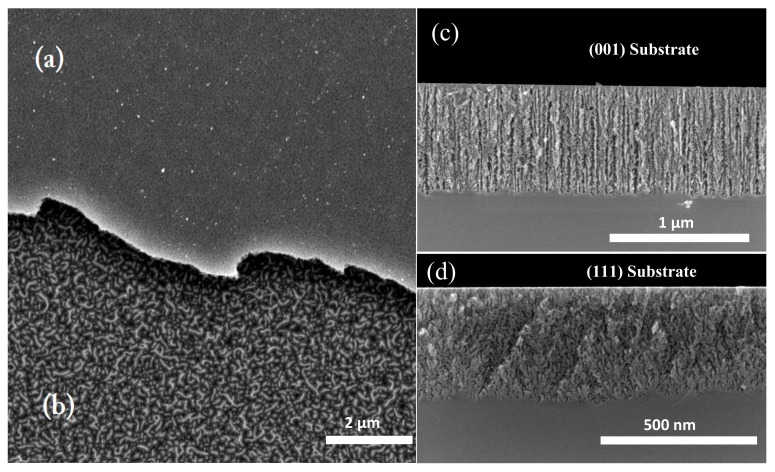
SEM images of the top view of the PSi double layer where (**a**) the first layer was anodized at 5 mA/cm${}^{2}$ and (**b**) the second one was anodized at 60 mA/cm${}^{2}$. Cross-section of single PSi films fabricated at 5 mA/cm${}^{2}$ using (**c**) (001) and (**d**) (111) substrate. The most critical features of PSi formation are its dependence on pore nucleation and growth of the substrate direction and the self-limited electrochemical reaction. That behavior allows to tune the refractive index of porous silicon and to fabricate multilayered structures.

Figure 1a,b show a top view SEM (scanning electron microscopy) image of a double layer of PSi where the first layer was fabricated at 5 mA/cm${}^{2}$ and the second one at 60 mA/cm${}^{2}$. There were no changes in the first layer porosity, and a new porous formation takes place under it. This self-limited process allows manufacturing PSi one-dimensional photonic crystals (PhC) such as distributed Bragg reflectors (DBR), omnidirectional mirrors (OMs) [6], and optical microcavities (OMC) [7], where their optical response is dependent on the photonic cell parameters such as porosity profile and thickness. This means that the design and fabrication of PSi PhC devices [8] require an accurate determination of optical constants as a function of porosity and etching kinetics. Then, it is possible to monitor and control the etching process using in situ techniques such as optical interferometry and photoacoustics [5,9,10,11]. Additionally, photoacoustics allows the characterization of the thermal properties of PSi structures and composites [12,13,14,15].

Light propagation in complex nanostructured materials is a field under constant development, and it is receiving increasing attention. Notably, (111)-PSi is a three-dimensional disordered network where its optical properties and architecture play a synergistic role. Usually, for PSi, the reflection and transmittance spectra are recorded to determine the optical constants (refractive index and extinction coefficient) through several methods (i.e., envelope method and Fresnel’s equation). However, these methods are limited in the study of highly absorbent and rough materials [16]. Even so, using near-specular reflectance, it is possible to obtain the optical constants, layer thickness, and surface roughness fitting the experimental spectrum using optimization procedures [1,17] and a suitable model.

In this direction, the PSi optical constants can be described in the visible and infrared range as a composite material using the effective medium approximation (EMA) [18,19]. This formalism is based on an average electrical permittivity description that depends on the incrustations fraction and its geometry giving rise to different rules such as the Maxwell Garnett [20,21], Bruggeman [18], and Looyenga [22].

Krzyżanowska et al. [23] study the refractive index versus porosity for single PSi layers obtained from p${}^{+}$type (111), finding that optical anisotropy increase as a function of porosity. Arenas et al. [24] develop a methodology to simultaneously calculate the complex refractive index and thickness of PSi films from their normal reflectance spectra without transmission information spectra finding that the absorption coefficient of the PSi sample over the Si substrate is close to the same free-standing sample. Specular reflectance measurements in PSi films and heterostructures show optical losses due to the scattering at the air-porous silicon interfaces [1].

**Figure 2:**
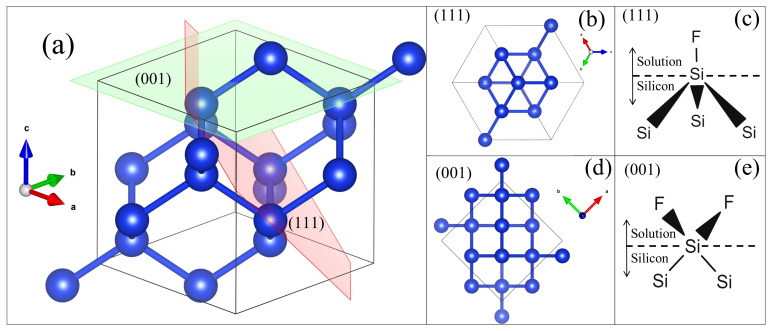
(**a**) Shows the crystalline unit cell of silicon and the (001) and (111) planes. Show crystalline cell since (**b**) (111) and (**d**) (001) planes. (**c**,**e**) are a molecular schematic of interface solution/Si for (111) and (001) planes respectively.

Although optical properties of PSi were studied from diverse theoretical solutions, for PSi obtained from (111) Si and others directions, the optical constants have been slightly studied. This is an essential fact because PSi exhibits a diverse porous morphology that depends on doping type, doping dose, substrate crystalline orientation [3,25] as Figure 1c,d show. In these figures, it is outstanding that the pore geometry changed as a function of substrate orientation. For <111> direction, pores are not straight aligned, exhibit branching and the etching rate is lower than <001>, even if the electrical characteristics are the same (i.e., doping dose and type) [26,27]. This behavior can be explained from a crystallographic point of view. Figure 2a shows the Si cell where (001) and (111) planes are indicated. In the case of <111> direction, each Si atom is bonded by one at the top and three at the bottom as is showed in Figure 2b. This means that during the etching, the superficial Si atoms only can be bonded by one F atom (Figure 2c). For <001> direction, each Si atom is bonded by two at the top and two and the bottom, as shown in Figure 2d. Thus each superficial Si can be bonded with two F atoms during the etching (Figure 2e). Therefore, Si dilution is favored in the <001> direction.

For this reason, in this work, optical constants of single (111) PSi layers as a function of current density and porosity are studied, determined, and compared using theoretical and experimental methods. The first method is based on in situ photoacoustic and scanning electron microscopy (SEM), which allows the refractive index direct determination using the antireflection condition in the photoacoustic amplitude [9]. In situ photoacoustic can determine the critical current when electropolishing occurs. The second method consists of a reflectance spectrum fitting using genetic algorithms [1] optimization procedure using different EMA rules and transfer matrix method (TMM).

## 2. Theoretical Models

### 2.1. A Collection of Mixing Rules

The electrical and optical properties of PSi thin films can be described using EMA rules. These rules define electrical permittivity as a mixture of a host material and a fraction of its incrustations. The most used binary rules to describe PSi are Maxwell Garnett (MG) [20,21], Bruggeman [18] (BR), and Landau-Looyenga-Lifshitz (LLL) [22]. In the MG model, the incrustation particles are spheres with low volumetric content, specific polarizability on either a lattice or randomly distributed in a fixed volume (dilute spheres). The BR formula is symmetric if the components are interchanged, which leads to better performance outside of the dilute limit. The BR model also represents a polynomial equation of order N, where N is the mixture’s number of components. This means that there are N solutions (possible degenerate) where only one represents the physical solution. In that case, the imaginary part of the effective dielectric function must be positive [20].

The LLL rule considers mixtures of irregular shapes particles with slight electrical contrast. Equations (1)–(3) describe the effective dielectric function for each of the models mentioned earlier.

(1) $$\left(\frac{{\hat{\epsilon }}_{ef}-{\hat{\epsilon }}_{M}}{{\hat{\epsilon }}_{ef}+2{\hat{\epsilon }}_{M}}\right)=p\left(\frac{{\hat{\epsilon }}_{i}-{\hat{\epsilon }}_{M}}{{\hat{\epsilon }}_{i}+2{\hat{\epsilon }}_{M}}\right),\;\;\;\;\left(\mathrm{MG}\right)$$

(2) $$\left(1-p\right)\left(\frac{{\hat{\epsilon }}_{M}-{\hat{\epsilon }}_{ef}}{{\hat{\epsilon }}_{M}+2{\hat{\epsilon }}_{ef}}\right)=-p\left(\frac{{\hat{\epsilon }}_{i}-{\hat{\epsilon }}_{e}f}{{\hat{\epsilon }}_{i}+2{\hat{\epsilon }}_{ef}}\right),\;\;\;\;\left(\mathrm{BR}\right)$$

(3) $${\left({\hat{\epsilon }}_{ef}\right)}^{1/3}=p{\left({\hat{\epsilon }}_{i}\right)}^{1/3}+\left(1-p\right){\left({\hat{\epsilon }}_{M}\right)}^{1/3},\;\;\;\;\left(\mathrm{LLL}\right)$$

where ${\hat{\epsilon }}_{ef}$ is the effective dielectric constant of the mixture, ${\hat{\epsilon }}_{M}$ is the dielectric constant of the host material, in this case c-Si, ${\hat{\epsilon }}_{i}$ is the dielectric constant of the incrustations (air), and *p* is the volume fraction of the incrustations. Therefore, optical constants can be calculated in the linear regimen through effective dielectric function [1]. The numerical calculations were carried out using the Si and air refractive index values reported by Green [28] and Liping et al. [29], respectively.

### 2.2. Thin Film Calculations

Herein, transfer matrix method (TMM) is used to calculate the reflectance of the air/PSi/Si structure such as that shown in Figure 1c,d. It is considered a dispersive, absorbent, isotropic and nonmagnetic material with refractive index $\hat{\eta }\left(\lambda \right)=n\left(\lambda \right)+ik\left(\lambda \right)$. Each interface is characterized by an admittance matrix $W_{k-1,k}$ and phase matrix $U_{k}$ given by [30,31]:

(4) $$\begin{array}{c} W_{k-1,k}=\frac{c_{k-1,k}}{t_{kR}}\left[\begin{array}{cc} 1 & -r_{kL}\\ r_{kR} & t_{kR}t_{kL}-r_{kR}r_{kL} \end{array}\right], \end{array}$$

(5) $$U_{k}=\left[\begin{array}{cc} \exp(i\phi ) & 0\\ 0 & \exp(-i\phi ) \end{array}\right]=\left[\begin{array}{cc} \exp(\frac{2\pi i}{\lambda }{\hat{\eta }}_{k}d_{k}\cos\theta_{k}) & 0\\ 0 & \exp(-\frac{2\pi i}{\lambda }{\hat{\eta }}_{k}d_{k}\cos\theta_{k}) \end{array}\right],$$

where $r_{k,L,R}$ and $t_{k,L,R}$ are the modified Fresnel coefficients that introduce the interface rms roughness ($\sigma_{ij}$) (assuming $\sigma_{ij}\ll \lambda$) in order to deal with incoherent propagation due to thick substrates, $c_{k-1,k}$ is the polarization factor, $d_{k}$ is the layer thickness, and $\theta_{k}$ is the incidence angle [4,30]. Thus, for the structure Air/Psi/Si, the total transfer matrix is given by:

(6) $$\begin{array}{c} S=\left[\begin{array}{cc} s_{11} & s_{12}\\ s_{21} & s_{22} \end{array}\right]=W_{01}U_{1}W_{12}, \end{array}$$

and the reflectance can be determined from *S* components as:

(7) $$\begin{array}{c} R=rr^{*}=\left(\frac{s_{21}}{s_{11}}\right){\left(\frac{s_{21}}{s_{11}}\right)}^{*}. \end{array}$$

On the other hand, (111) PSi exhibits branched pores that produce high scattering observed in the attenuation of specular light intensity. Thus, the absorption coefficient can be described as a superposition of the film absorption $\left(\alpha_{true}\right)$ and the contribution of volume scattering $\left(\alpha_{s}\right)$ as:

(8) $$\begin{array}{ccc} \alpha_{f}\left(\lambda \right) & = & \alpha_{true}\left(\lambda \right)+\alpha_{s}\left(\lambda \right). \end{array}$$

Hence, effective extinction coefficient can be written as follows [32]:

(9) $$\begin{array}{ccc} k_{f}\left(\lambda \right) & = & k_{true}\left(\lambda \right)+\frac{\alpha_{s}\lambda }{4\pi }, \end{array}$$

Equations (6) and (7) represent the fitness function used in the genetic algorithms to fit the experimental reflectance and determine the porosity, thickness, scattering factor, and interface roughness by solving the following optimization problem comparing the measured $\left(R_{exp}\right)$ and analytical calculated reflectance $\left(R_{teo}\right)$ as follows:

(10) $$\begin{array}{c} F\left(\lambda_{i},p,d,\alpha_{s},\sigma \right)=\sum_{i=1}^{N}w\left(\lambda_{i}\right){\left[R_{exp}\left(\lambda_{i}\right)-R_{teo}\left(\lambda_{i},p,d,\alpha_{s},\sigma \right)\right]}^{2}, \end{array}$$

where *F* is the penalty function used as a criterion to select best solutions and represents the weighted misfit between the experimental and theoretical simulated spectrum, *N* is the total number of points of the experimental spectrum, and $w(\lambda_{1})$ is and weight factor to improve the convergence of the GA method. Therefore, the lower value of *F* represents the better solution. In this particular case, it is considered $w(\lambda_{1})$ for all spectral range.

### 2.3. In Situ Photoacoustics

In the case of in situ photoacoustics, the main mechanism of the acoustic signal generation is the heat conduction towards a gas cell, produced by the absorption of modulated light by a solid sample. Rosencwaig and Gersho (RG) model [33] allows determining the temperature field through the solution of the one-dimensional heat Equation (11),

(11) $$\begin{array}{ccc} \frac{\partial^{2}T\left(x,t\right)}{\partial x^{2}}-\frac{1}{\alpha }\frac{\partial T(x,t)}{\partial t} & = & \frac{\beta I_{0}\xi \left(1-R(t)\right)}{2\kappa_{s}}e^{\beta x}\left(1+e^{i\omega t}\right),\\ & \mathrm{for} & \;\;0<x\leq l_{Si}, \end{array}$$

where $I_{o}$ is the intensity of the light source and ω its modulation frequency, β is the absorption coefficient, ξ the efficiency of the light absorption, $\kappa_{s}$ the thermal conductivity, and R(t) the reflectance of the heterostructure, which is time-dependent. The spatial distribution along the multilayered structure can be obtained by solving the heat conduction equation (Equation (11)) with the appropriate mobile boundary conditions [10,34].

**Figure 3:**
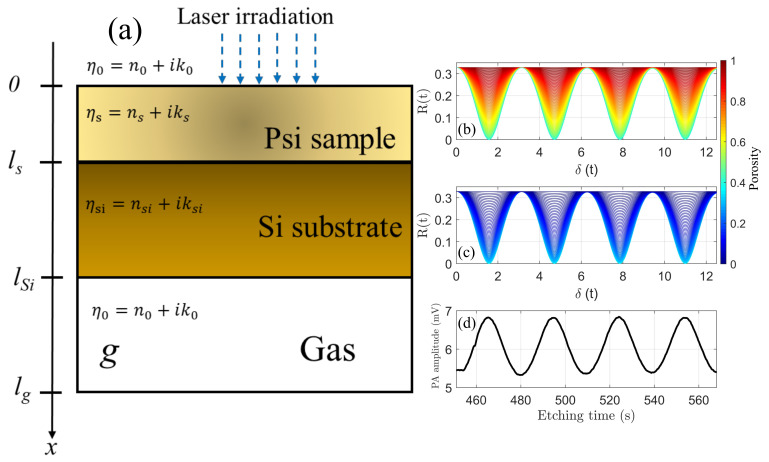
(**a**) Schematic sketch-view of an electrochemical cell coupled to a photoacoustic one during the PSi formation. (**b**,**c**) represent the reflectance of heterostructure as a function of the thickens of PSi Equation (13) and porosity. (**d**) is the PA amplitude as a function of the etching time for a sample anodized at 5 mA/cm${}^{2}$.

Figure 3a shows the schematic representation of the cell, where $l_{s}$ interface represents a mobile boundary condition. Ramirez-Gutierrez et al. [5,9,10] introduced a modification of the RG model considering a self-modulation effect due to thin-film interference given a reflectance function as follows:

(12) $$\begin{array}{c} R\left(t\right)=\left|r^{2}\right|=\frac{\cos^{2}\left(\delta \right){\left(\alpha_{0}-\alpha_{1}\right)}^{2}+\sin^{2}\left(\delta \right){\left(\alpha_{0}\alpha_{1}-1\right)}^{2}}{\cos^{2}\left(\delta \right){\left(\alpha_{0}+\alpha_{1}\right)}^{2}+\sin^{2}\left(\delta \right){\left(\alpha_{0}\alpha_{1}+1\right)}^{2}}, \end{array}$$

that depends on reflectance coefficient phase term, that at normal incidence is given by:

(13) $$\begin{array}{c} \delta \left(t,\lambda_{0},p\right)=\frac{2\pi d(t)}{\lambda_{0}}n_{s}\left(\lambda_{0},p\right), \end{array}$$

where $\lambda_{0}$ is the laser source wavelength, $\alpha_{0}=n_{0}/n_{s}$ and $\alpha_{1}=n_{Si}/n_{s}$ are refractive index ratio, $d\left(t\right)=v_{etch}t$ is the PSi film thickens; that can be expressed as a function of etching rate ($v_{etch}$) and the etching time (*t*), and $n_{s}\left(\lambda_{0},p\right)$ is the real part refractive index at $\lambda_{0}$ that is also porosity-dependent.

From a physics point of view, Equation (11) is an equation that contends, in our case, two thermal sources. The first term Equation (12) is related to the interaction between the incident and the reflected beam that are thickness- and refractive-index-dependent, and it is the base of the photoacoustic detection due to the interference effect as can be seen in Figure 3b,c and reported elsewhere [9]. The second term in Equation (11) corresponds to the modulated incident radiation that is modulated at ω and absorbed by the sample. Sensing the PSi formation, light modulation (ω) is fixed to guarantee the thermally thin regime to obtain information along the heterostructure. This term contributes to the DC level of the PA amplitude signal.

When the electrochemical reaction is on, the PSi film length increases, producing an interference effect. Thus the reflectance (R(t)) varies in time as a function of PSi thickness. Therefore, this equation sense in real time the changes in the optical path and give information on the kinetics of the etching.

The solution in the time domain allows the calculation of the instantaneous PSi etching rate and thermal properties [13]. However, in this experimental configuration, light modulation frequency (ω) remains constant during the etching monitoring. Therefore the variation in the PA amplitude is mainly due to the interference effect of thin films. Since the voltage detected from the microphone is proportional to the reflectance changes from the sample, it is possible to correlate the PA amplitude with its optical properties (refractive index).

Reflectance in Equation (12) is a periodic function that changes continuously in the time [9] and has maximums (reflective) and minimums (antireflective) when $\delta =\left(m-\frac{1}{2}\right)\pi$ and δ=mπ, respectively, as it is shown in Figure 3b,c. The parameter m≥1 is an integer number representing the periods of reflectance in which the minimum and the maximum of reflectance is associated with the maximum and minimum of PA amplitude, respectively [7,9]. Thus, the refractive index can be obtained from Equation (13) if the thickness is known and PA amplitude ends in a local maximum or minimum where the value of (δ) is known [7,9]. To determine the PSi etching rate and thermal properties, it is mandatory to solve the heat conduction equation reported elsewhere in Ref. [10].

## 3. Experimental Section

### 3.1. Porous Silicon Fabrication

A p-type Si with 0.01 Ω cm of resistivity and (111) orientation from the Polishing Corporation of America (Santa Clara, CA, United States) was used to fabricate fifteen samples of PSi as a function of anodizing current density from 5 to 110 mA/cm${}^{2}$. Table 1 summarizes the anodizing conditions for each sample. The samples were cleaved along (110) plane using a diamond tip scribe to scratch the wafer and a pair of tweezers to bend and cleave it in small parallelograms of a=b=14 mm and $\alpha =60^{\circ }$. All samples were cleaned using RCA standard method for organic removal. For the electrical circuit, a copper foil was used as an electrical contact with the Si sample and a platinum foil as a counter electrode. Both electrodes were connected to a precision current source Keithley 6220 (Tektronix, Beaverton, OR, United States). The electrolyte was an HF (40%) (J.T. Baker, Deventer, Netherlands)/EtOH (99.67%) (Sigma-Aldrich, Toluca, Mexico) solution in a 3:7 volume ratio, and etching was carried out using an electrochemical cell coupled to a photoacoustic one to monitor in real-time the PSi film formation [5,9]. It was fabricated 15 PSi samples covering current densities from 5 to 110 mA/cm ${}^{2}$ and anodized during four photoacoustic cycles.

#### Reflectance Spectrometry

A Perkin Elmer UV-Vis Spectrophotometer Lambda 35 in the near normal (6${}^{\circ }$) configuration was used to measure the absolute reflectance [1] of PSi films. The equipment was self-calibrated using an aluminum mirror from 1100 to 210 nm. A polished Si wafer was used as a reference to obtain absolute reflectance [1].

## 4. Results

### 4.1. In Situ Photoacoustic

It was used the differential photoacoustic setup publish previously by Ramirez-Gutierrez et al. [7] to monitor the PSi formation in real-time. The Si sample seals the electrochemical cell, and a capacitive microphone located at the backside records the sound signal generated by the light absorption and electrochemical reaction. The photoacoustic (PA) signal is generated using a laser (Laser-Mate Group, Walnut, CA, USA) of 808 nm wavelength and <200 mW, modulated at 23 Hz. That frequency was selected in order to guaranty the thermally thick regimen. The PA amplitude signal is periodic and depends on the current density; consequently, four PA cycles were selected as an etching time for each current density. The PA phase is not sensible because all monitoring process is carried out at constant frequency.

**Figure 4:**
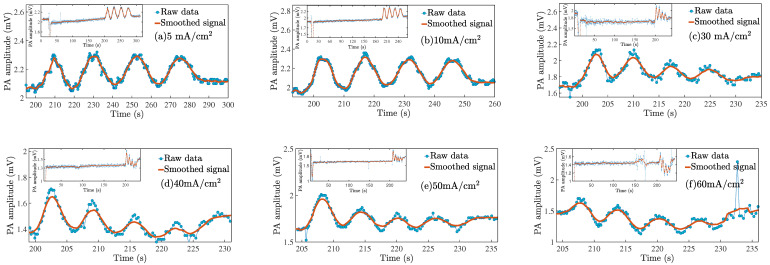
Show the in situ photoacoustic amplitude recorded during the etching process. Each graph corresponds to a specific current density for the samples summarized in Table 1. The PA amplitude changes periodically when the current injection is on. The insets show the complete anodizing history.

**Figure A1:**
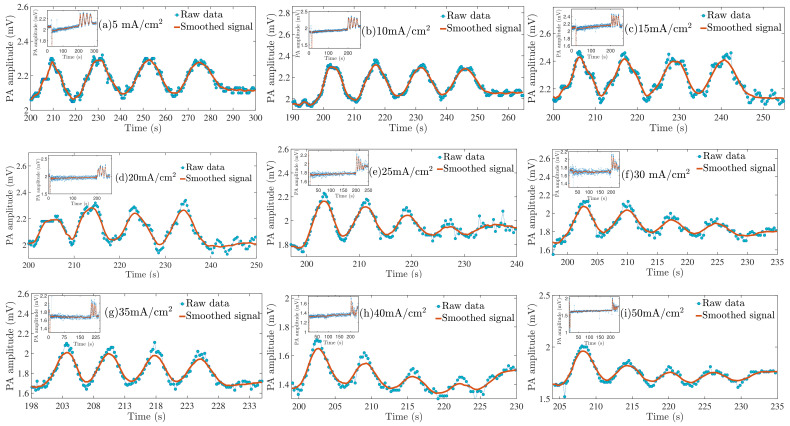

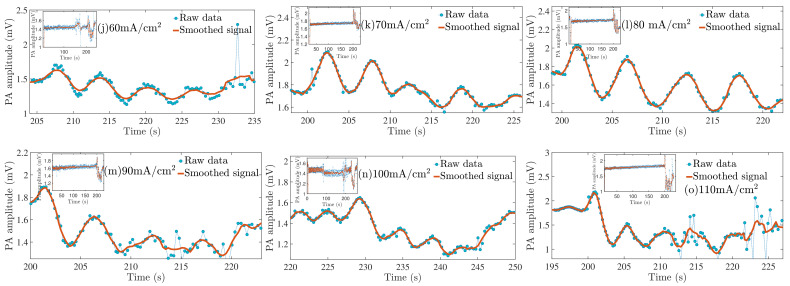
Show the in situ photoacoustic amplitude recorded during the etching process. Each graph corresponds to a specific current density for the samples summarized in Table 1. The PA amplitude changes periodically at the moment of the current injection due to thin-film interference between PSi and Si substrate. The insets show the complete anodizing history.

Figure 4 shows the PA amplitude as a function of time for six representative samples of the all series samples (from 5 mA/cm${}^{2}$ to 110 mA/cm${}^{2}$, show Figure A1). The in-set in every graph shows the complete PA signal during all PSi fabrication processes. First, a perturbation of PA amplitude takes place due to electrolyte emptying. It is then waiting for about 200 seconds to guarantee the silicon oxide removal from the sample surface. Finally, an oscillation of the PA signal occurs when the etch starts due to the current injection. For Figure 4a (5 mA/cm${}^{2}$) to Figure 4f (60 mA/cm${}^{2}$), the PA signal period is well-defined that is an indicative porous layer that allows the interference effect. The current source is turned off when four PA cycles are reached, and of course, the PA signal return to be constant. Particularly, in Figure A1k (70 mA/cm${}^{2}$) to Figure A1o (110 mA/cm${}^{2}$), the PA amplitude baseline exhibits a drop that is indicative of an electropolishing regimen. Therefore, Equation (13) is not valid to determine the refractive index (see Appendix A). However, the shape of the PA amplitude indicates if there is pore film-forming or an electropolishing occurs.

**Figure 5:**
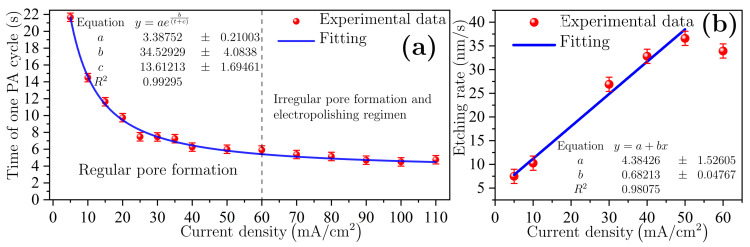
(**a**) Time of one PA cycle as a function of current density. (**b**) Etching rate in the linear regimen (J<60 mA/cm${}^{2}$).

The data extracted from anodizing history determined the value of one PA cycle for each current density that determines the reaction kinetics. Figure 5a shows the value of one PA cycle as a function of current density. As can be seen, PA cycle time exhibits an exponential decay behavior because the current increases the etching rate and porosity too. Therefore, interference conditions occur every time in less time. Typical anodic J−V relationship for Si in HF media showing three specific regions: first is a regular pore formation where *J* is exponentially proportional to *V*. Then, there is a transition zone where non-regular pore formation takes place. Finally, a maximum of *J* is reached, which indicates the Si electropolishing zone [35]. In this case, PA signals in Figure 4 show that the transition zone near to electropolishing regimen stars from 60 mA/cm${}^{2}$, and the data fitting allow predicting the PA cycle time for any *J* value in the pore formation region. This behavior can be evidenced in Figure 5b, where the etching rate as a function of current density for J<60 mA/cm${}^{2}$ is a linear function. After, the point goes out of trend.

### 4.2. Morphological Characterization

**Figure 6:**
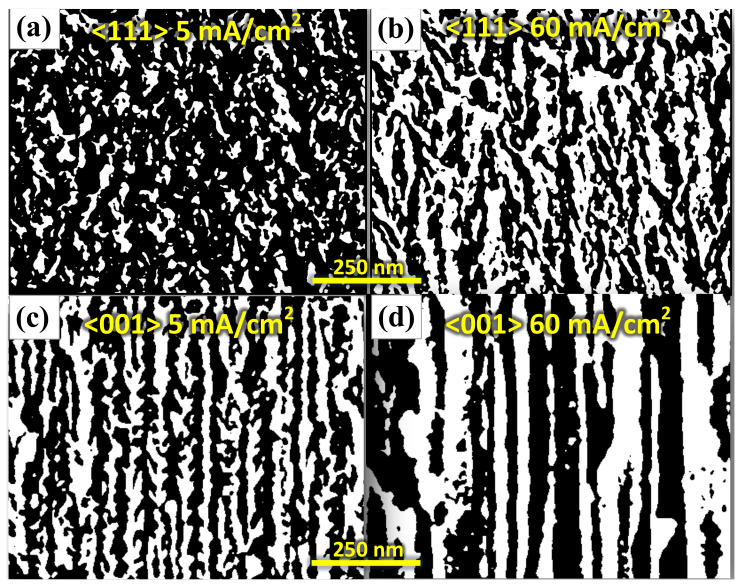
Contrasted SEM images for PSi (**a**) (111) at 5 mA/cm${}^{2}$, (**b**) (111) at 60 mA/cm${}^{2}$, (**c**) (001) at 5 mA/cm${}^{2}$, and (**d**) (001) at 60 mA/cm${}^{2}$. SEM images were treated and analyzed with ImageJ software [36].

Pore growth is strongly dependent on substrate crystalline orientation. Specifically, the etching rate is slower in (111) than (001) substrates. This effect can be evidenced in Figure 6, where it shows a contrasted SEM image of the cross-section of (111) PSi compared to (001) PSi fabricated under the same conditions. Figure 6a,b exhibit more significant density than Figure 6c,d, which means that porosity reached by (111) substrates is less than (001). The pore branching for (111) direction is more remarkable, while in the case of (001), pores are straight aligned.

**Figure 7:**
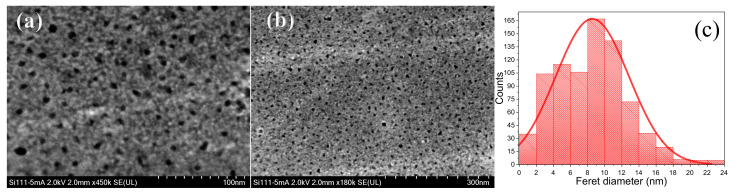
(**a**,**b**) PSi surface SEM image of PSi fabricated at 5mA/cm${}^{2}$. (**c**) Porous size distribution obtained by SEM images analysis using ParticleSizer by ImageJ software (open source) [36]. Distance in pixels is fixed using the reference bar of the SEM image. Then, a good part of the SEM image is selected for size analyzes. ImageJ threshold tool is used to set manually lower and upper lightness threshold values segmenting grayscale images into features of interest and background. If it is requiring, a bandpass filter can be applied to improve the porous edge determination. Finally, porous edges are created by tracing objects of uniform color or thresholded objects. Particle or pore size of thresholded images can be extracted by specifying the suitable edge ranges and choosing if their outer edge or flood filling should trace particles.

Figure 7a,b show a top view of the S5 (5 mA/cm${}^{2}$) sample in which evenly distributed pores can be seen. The pore size (Feret diameter) follows a normal distribution centered about 8.5 nm. Pore diameter, as well as the porosity, increases as current density increases. Meanwhile, the pore distribution regularity decreases [37,38].

**Figure 8:**
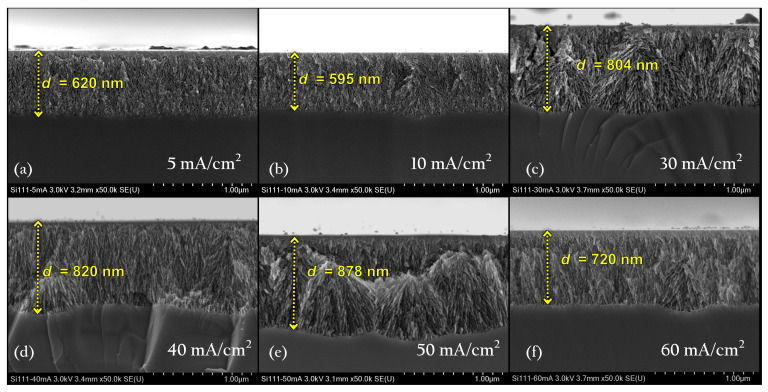
Cross section SEM images of PSi films at different anodizing current densities. (**a**) 5 mA/cm${}^{2}$, (**b**) 10 mA/cm${}^{2}$, (**c**) 30 mA/cm${}^{2}$, (**d**) 40 mA/cm${}^{2}$, (**e**) 50 mA/cm${}^{2}$, and (**f**) 60 mA/cm${}^{2}$. The micrographs show the interface between the PSi film and bulk.

Figure 8 shows the cross-section scanning electron microscopy (SEM) images of some representative samples. It is crucial to notice that anodizing time is not the same for each sample because the control parameter is the photoacoustic cycle related to the refractive index and thickness. As Figure 5 shows, the PA cycle decreases as a function of current density, which means that the anodizing time was shorter as the current density increases. Accordingly, the thickness of the sample is similar between them. Nevertheless, the system shows a few general trends: under these anodizing conditions, mesoporous films are obtained, the pores are laterally branched, and the pore diameter or porosity increases with increasing current density and the etching rate.

For low current densities (J<30 mA/cm${}^{2}$), PSi films look dense, and the interface with the Si substrate is moderately smooth. With increasing the current density, there appear zones of branching nucleation that look like fir-tree type structure randomly oriented and interconnected. This particular behavior produces evident undulations in the interface PSi/substrate that can be interpreted as increasing the roughness at those interfaces. This effect may have been generated by the pores’ branching that makes diffusion of the electrolyte through the film more difficult as the etching rate increases. Therefore, pores formation becomes time-dependent [25] explaining the deviations of some experiments. It means that each experiment or sample anodizing is different even though the same conditions have been fixed. Intrinsic parameters like carrier distribution, cutting damage, or extrinsic factors such as electrolyte temperature and electrolyte diffusion impact the final result.

### 4.3. Optical Characterization

It was used three different EMA rules, the TMM method, and the genetic algorithm to determine the best fit with UV-Vis experimental reflectance spectrum to describe optical constants of (111) Psi. The reflectance is measured at 6${}^{\circ }$ of incidence with no polarized light. Therefore, the theoretical calculation considered arithmetic mean between s- and p-polarized light.

**Figure 9:**
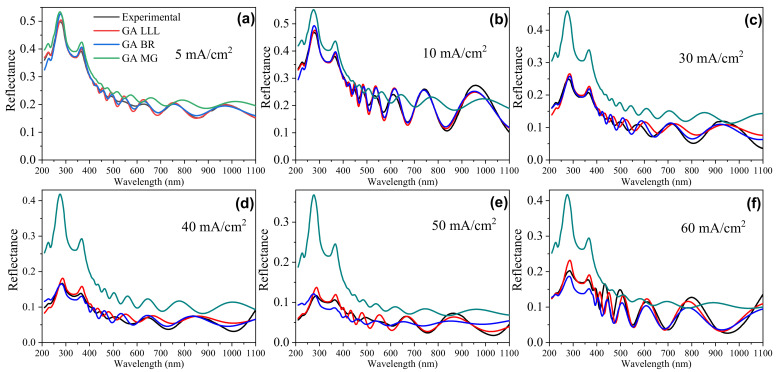
Near normal incidence reflectance (6${}^{\circ }$) of representative samples as a function of wavelength. (**a**) 5 mA/cm${}^{2}$, (**b**) 10 mA/cm${}^{2}$, (**c**) 30 mA/cm${}^{2}$, (**d**) 40 mA/cm${}^{2}$, (**e**) 50 mA/cm${}^{2}$, and (**f**) 60 mA/cm${}^{2}$. The measured reflectance is compared to the theoretical reflectance calculated by Looyenga (LLL), Bruggeman (BR), and Maxwell Garnett EMA rules. The best fit value of porosity, thickness, absorption coefficient, and interface roughness are reported in Table 2.

The implemented genetic algorithm minimizes the difference between the theoretical and experimental reflectance curves to optimize porous layer parameters. As Equation (10) shows, the optimized parameters are $\left(p,d,\alpha_{s},\sigma_{ij}\right)$. As spectra in Figure 9 show, the specular reflectance intensity is lower than that reported for (001) analog films. Consequently, it is assumed an additional absorption due to bulk scattering Equation (9).

Figure 9 shows the comparison between the experimental and theoretical reflectance calculated by each EMA rule. It was observed that Maxwell Garnett (MG) approximation does not fit properly. This is because the MG rule is designed for a dilute regimen, and this is not the case according to microscopy and photoacoustic calculation where porosity value is more significant than 30%. Furthermore, as the anodizing current increases, the porosity increases, and the approach worsens.

In BR and LLL approximations, a good fit between experimental and theoretical spectra was obtained. However, the best fit is obtained using the LLL rule. Despite that, there is not a completed match between the experimental and simulated spectrum because this kind of film presents an optical anisotropy [23,39] due to particular porous geometry (Figure 8e, and birefringence [40,41,42] that the model does not take into account.

**Figure 10:**
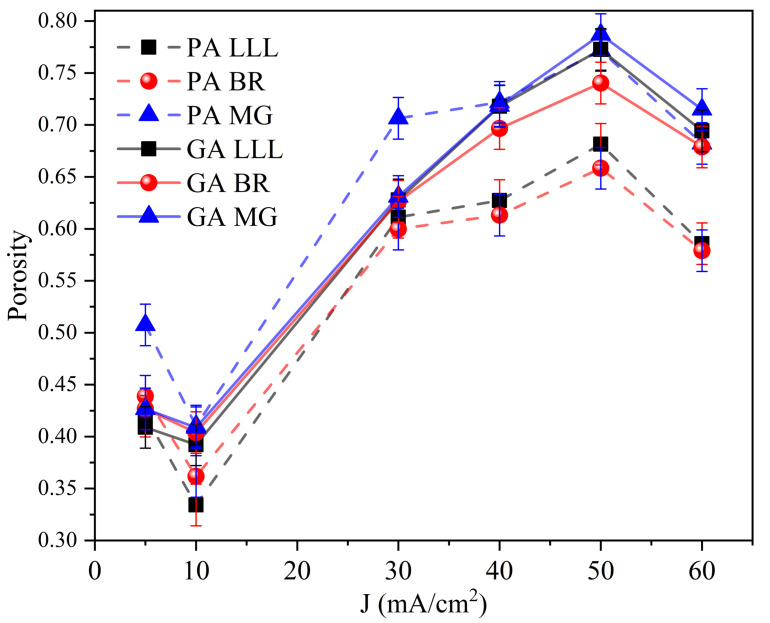
Porosity as a function of current density estimated by EMA rules combined with in situ photoacoustic (PA) and genetic algorithms (GA).

The porosity determination of the PSi films is of great importance for the design and fabrication of photonic devices based on PSi. Figure 10 and Table 2 summarize the porosity values as a function of current density obtained through genetic algorithms (GA) compared to those determined by in situ photoacoustic (PA). In contrast, there is a good agreement among the methods for low current densities. There is a discrepancy for values from *p* to *J* greater than 30 mA/cm${}^{2}$ because pore formation can stay in the transition region close to the electropolishing regimen where the anisotropy of the material increases. In this sense, the value obtained for *p* through PA can be closer to the real (measured by porosimetry or gravimetry) since the estimate is made in real-time and even with normal light incidence minimizing the anisotropy effects. This means that the refractive index estimated by the porosity and the EMA rule corresponds to the ordinary.

Figure 10 shows the porosity as a function of current density calculated through different methods. The *p* value obtained is model-dependent; however, it has the same behavior. The porosity of the PSi layer increases with current density at this given electrolyte concentration. However, for 10 and 60 mA/cm${}^{2}$ the value falls. This is not a general trend for (111) PSi but is due to local variations in substrate resistivity since the boron concentration is not uniform [7] or cutting damage.

## 5. Conclusions

There are many papers regarding PSi fabrication processes and summarizing data of porosity and optical properties behavior. However, there is low repeatability among the results of different experiments obtained using the same experimental setup because of multiple parameters involve during the Si anodizing. Therefore, real-time monitoring techniques, such as photoacoustics, are crucial because they can avoid this issue allowing feedback during the fabrication process. For (111) PSI formation, PA amplitude shape indicates regular porous film formation. Well-defined cycles should be displayed whose amplitude decreases as the film thickens. Any deviation in the PA amplitude period or intensity indicates a fluctuation in the etching process or a kinetic change in the chemical reaction. This information can be used to implement a complete control to compensate in real-time that deviations.

Porous silicon (111) obtained through electrochemical anodizing forms denser structures than for PSi (001), and it is possible to get films with low porosities below 30%. These branched films produce more light scattering, which was quantified through the addition of the scattering absorption coefficient. Effectively, these optical losses can be characterized as effective absorption by separating the absorption coefficient and scattering contributions. In situ photoacoustics can be used synergistically with the genetic algorithms and specular reflectance to determine the accurate porosity value without obtaining the thickness obtained through SEM, allowing to build an experimental dispersion relationship that does not depend on some model of the effective medium. Finally, (111) PSi can be a good prototype material for highly scattering films for structural color applications or reflectors.

## Figures and Tables

**Table 1:** Anodizing conditions of PSi samples. The anodizing time reduces monotonously as a function of current density. All samples were etched during four photoacoustic cycles.

Sample Name	J±0.01 (mA/cm${}^{2}$)	$t_{\mathrm{etch}}\pm 0.1$ (s)	*d* (nm)
S5	5	86.6	620
S10	10	58.0	595
S15	15	46.5	–
S20	20	39.0	–
S25	25	29.9	–
S30	30	29.8	804
S35	35	29.8	–
S40	40	25.0	820
S50	50	24.0	878
S60	60	23.6	720
S70	70	21.4	–
S80	80	20.5	–
S90	90	18.8	–
S100	100	18.0	–
S110	110	19.0	–

**Table 2:** Experimentally observed and numerically optimized values for the porosity (p), thickness (d), absorption coefficient $\left(\alpha_{s}\right)$, and roughness (σ) using effective media approximation combined with genetic algorithms and in situ photoacoustics.

Sample	EMA Rule	p±0.02	d±10 nm	$\alpha_{s}\pm 0.1$ cm ${}^{-1}$	σ±1 nm
S5	LLL	0.40	622	3.3	5
	BR	0.42	615	3.1	7
	MG	0.42	606	3.4	9
	PA	0.41 ${}^{†}$	620 *	-	-
S10	LLL	0.39	589	1.0	6
	BR	0.40	577	0.9	9
	MG	0.40	577	2.6	8
	PA	0.33 ${}^{†}$	595 *	-	-
S30	LLL	0.62	784	3.0	9
	BR	0.62	783	2.6	3
	MG	0.63	814	2.8	10
	PA	0.61 ${}^{†}$	804 *	-	-
S40	LLL	0.71	810	3.5	10
	BR	0.69	802	3.1	3
	MG	0.71	821	2.8	9
	PA	0.62 ${}^{†}$	820 *		
S50	LLL	0.77	888	2.5	8
	BR	0.74	889	3.9	3
	MG	0.78	874	3.5	10
	PA	0.68 ${}^{†}$	878 *	-	-
S60	LLL	0.69	704	2.1	3
	BR	0.67	723	2.4	1
	MG	0.71	722	4.2	10
	PA	0.58 ${}^{†}$	720 *	-	-

^†^ photoacoustic determination; * SEM determination.

## Data Availability

The data presented in this study are available on request from the corresponding author.
